# Prognostic factors affecting the risk of thoracic progression in extensive-stage small cell lung cancer

**DOI:** 10.1186/s12885-016-2222-4

**Published:** 2016-03-08

**Authors:** Tomoya Fukui, Michiko Itabashi, Mikiko Ishihara, Yasuhiro Hiyoshi, Masashi Kasajima, Satoshi Igawa, Jiichiro Sasaki, Noriyuki Masuda

**Affiliations:** Department of Respiratory Medicine, Kitasato University School of Medicine, 1-15-1 Kitasato, Minami-ku, Sagamihara, Kanagawa 252-0374 Japan; Research and Development Center for New Medical Frontiers, Kitasato University School of Medicine, 1-15-1 Kitasato, Minami-ku, Sagamihara, Kanagawa 252-0374 Japan

**Keywords:** Small-cell lung cancer, Extensive-stage, Prognostic factor, Thoracic progression

## Abstract

**Background:**

The efficacy of combined modality therapy is evaluated for patients with extensive-stage (ES) small cell lung cancer (SCLC). This study evaluated prognostic factors affecting the risk of thoracic progression in ES-SCLC patients likely to undergo thoracic radiotherapy combined chemotherapy.

**Methods:**

A retrospective review of ES-SCLC patients who had received systemic chemotherapy at our hospital was performed. Tumor size, metastatic sites, and laboratory data at diagnosis were evaluated as potential prognostic factors. In ES-SCLC patients without pleural dissemination, the rate of thoracic progression after initial chemotherapy was assessed.

**Results:**

Eighty-three of 96 consecutive ES-SCLC patients were analyzed. The overall response rate was 55 %, median progression free survival was 5.0 months (mo), and overall survival (OS) was 9.2 mo. Tumor size (19.4 mo for ≤3 cm vs. 8.5 mo for >3 cm, *p* = 0.017) and the number of metastatic sites (12.9 mo for single sites vs. 7.1 mo for multiple sites, *p* = 0.015) were prognostic factors, in addition to known prognostic factors such as performance status and the levels of LDH and sodium. Cox proportional hazard model showed that the OS was significantly worse in patients with large (>3 cm) primary tumor size {HR 2.44 [95 % confidential interval (CI) 1.05–5.68], *p* = 0.038} and multiple metastatic sites [HR 1.81 (95 % CI 1.08–3.04), *p* = 0.026]. In 51 cases without pleural dissemination, the number of metastatic sites was associated with thoracic progression after initial chemotherapy (65 % for single sites vs. 36 % for multiple sites, *p* = 0.036).

**Conclusion:**

Large tumor size and multiple metastatic sites at diagnosis significantly predicted poor survival in ES-SCLC patients. Based on the high rate of thoracic progression in ES-SCLC patients with single site of distant metastasis, we should consider thoracic radiotherapy combined with chemotherapy for this population.

**Electronic supplementary material:**

The online version of this article (doi:10.1186/s12885-016-2222-4) contains supplementary material, which is available to authorized users.

## Background

Small cell lung cancer (SCLC) is an aggressive disease characterized by a high propensity for metastases and a poor prognosis [1, 2]. SCLC is clinically categorized according to the disease extent as “limited-stage (LS)” defined as disease that can be encompassed within a tolerable radiation field and “extensive-stage (ES)” defined as outside of these confines [3]. In LS-SCLC patients, the addition of early concurrent thoracic radiotherapy (TRT) to chemotherapy has improved long-term survival [3–5]. Furthermore, prophylactic cranial irradiation (PCI) following complete or near-complete response after induction chemoradiotherapy provided additional survival benefit [3, 6].

ES-SCLC is a metastatic disease [1]. The standard treatment against ES-SCLC is combination chemotherapy, generally platinum plus etoposide or irinotecan [7–9]. Regardless of high sensitivity for initial chemotherapy, the survival of ES-SCLC patients has improved little in recent decades, showing 8–13 months (mo) of median survival time (MST) with less than 5 % surviving for two years [1, 10–12]. Recently, combined-modality therapy was evaluated in ES-SCLC patients. In a European study, PCI reduced the risk of brain metastases and prolonged the overall survival (OS) of ES-SCLC patients [13]. However, in a second trial that was conducted in Japan, the efficacy of PCI in ES-SCLC patients was controversial and the trial was stopped prematurely for futility [14]. Next, the use of TRT in addition to PCI following systemic chemotherapy was evaluated because intra-thoracic progression was common in the European trial for ES-SCLC patients and 75 % of the patients had persisting intra-thoracic disease after chemotherapy. This study suggested that ES-SCLC patients who respond to chemotherapy should be considered for TRT added to PCI with survival benefit; however, OS at 1 year, which was the primary endpoint in the study by Slotman, was not significantly improved (HR 0.84; 95 % CI: 0.69–1.01; *p* = 0.066) [15]. We surmised that the results indicated heterogeneity in the ES-SCLC patients and some of these patients might benefit from TRT.

To further improve the therapeutic efficacy, we hypothesized that TRT combined with chemotherapy should be considered for selected ES-SCLC patients. We identified the potential prognostic factors in ES-SCLC patients in addition to the well-established factors of age, gender, PS, serum LDH levels, and sodium levels [1]; we retrospectively evaluated ES-SCLC patients treated with systemic chemotherapy for identifying appropriate patients likely undergo TRT combined chemotherapy.

## Methods

### Study patients and clinical data collection

We retrospectively analyzed consecutive ES-SCLC patients with who had been pathologically diagnosed with SCLC and began first-line systemic chemotherapy between July 2008 and December 2012 at the Kitasato University hospital (Kanagawa, Japan). ES-SCLC was defined as a disease with distant metastases over one radiation field. ES-SCLC patients who had no laboratory and radiologic data before treatment and had a history of prior or concurrent other malignancy were excluded. The maximum tumor size of primary lesions using CT imaging, the sites (organs) of metastatic lesions, laboratory data, including level of Alb, ALP, LDH, sodium, CEA, NSE, and pro-GRP before treatment were evaluated as candidates of prognostic factors. After the pretreatment evaluation, including blood tests, chest X-rays, chest and abdominal CT scans, cranial CT scans or MRIs, and PET or bone scintigrams, chemotherapy was initiated for ES-SCLC. The patients’ responses were typically assessed after 2–4 cycles of systemic chemotherapy, and the patients returned for follow-up visits at our hospital each month. If recurrence was suspected, additional examinations such as CTs and MRIs were performed in accordance with their doctor’s instructions. OS was defined as the time from the start of first-line chemotherapy until death (data cut-off: September 30, 2014). The retrospective study was approved by the Kitasato University Medical Ethics Organization (B15-78), which waived the requirement for patients’ informed consent.

### Patient characteristics

We identified a total of 96 ES-SCLC patients who were initially treated with systemic chemotherapy between January 2009 and December 2012. We excluded four patients with double cancers and nine patients who were not evaluated by CT imaging before first-line chemotherapy. The analyzed characteristics of the 83 patients are listed in Table 1. The median size of the primary tumor was 5.4 cm (range 1.2–10.8 cm). Thirty-two of the 83 patients (39 %) had pleural dissemination at diagnosis and 3 (4 %) had brain metastases. In 36 patients (43 %), the ES-SCLC had spread to a distant organ, and in the remaining 47 patients (57 %), it had metastasized to multiple sites (range 2–5 sites), excluding the hilar, mediastinal, and subclavicular lymph nodes.

**Table 1:** Patient characteristics in this study

	ES-SCLC (*n* = 83)	%
Age, median (range), years	71 (47–91)	
<75 years	60	72
≥75 years	23	28
Gender		
Male/Female	73/10	88/12
Smoking status		
Never	2	2
Former/Current	49/30	59/36
Unknown	2	2
WHO performance status		
0/1	11/48	13/58
2/3/4	13/9/2	16/11/2
Size of primary tumor, median (range), cm	54 (12–108)	
≤3 cm	10	12
>3 cm	73	88
Extensive disease		
Intrathoracic disease	23	28
Distant (extrathoracic) metastases	29	35
Both	31	37
Sites of metastases		
Brain	3	4
Lung	28	34
Liver	29	35
Adrenal grand	11	13
Bone	25	30
Pleural dissemination	32	39
Others^a^	25	30
Number of metastatic sites (organs)		
Single (1)	36	43
Multiple (2/3/4/5)	23/17/5/2	28/20/6/2

^a^Including, 10 abdominal and 4 cervical lymph nodes, 4 pancreas, 4 bone marrow, 3 soft tissue, 1 pericardia, 1 parotid, and 1 ovary metastases

### Statistical analyses

All survival analyses were performed using the Kaplan–Meier method. The survival between the subgroups based on prognostic factors was compared using a log-rank test. Multivariate analysis was performed using a Cox proportional hazard model. The impact of the prognostic factors for the risk of thoracic progression was assessed using Pearson κ^2^ test. All analyses were performed using the SPSS statistical package (SPSS Inc, Chicago, IL).

## Results

### Effects of chemotherapy

Thirty-three patients (40 %) were treated with platinum-based combination chemotherapy and 41 patients (49 %) were given amrubicin monotherapy. Sixty-two patients (74 %) received three or more cycles of first-line chemotherapy, and 54 patients (65 %) received second-line or more chemotherapy after relapse. The overall response rate was 55 % (55/83) and the median progression free survival (PFS) was 5.0 mo (95 % CI: 4.5–5.5 mo). Twenty-three patients (28 %) relapsed after three mo from the completion of the initial therapy (sensitive relapse). The OS was 9.2 mo (95 % CI: 7.6–10.2 mo) in total cohort: 19.4 mo (95 % CI: 12.9–25.9 mo) for the sensitive group and 6.9 mo (95 % CI: 6.3–7.6 mo) for the refractory group (Table 2).

**Table 2:** Effects of First-line Chemotherapy for Patients with ES-SCLC

	ES-SCLC (*n* = 83)	%
Regimen		
CDDP + CPT	5	6
CDDP + VP-16	3	4
CBDCA + VP-16	20	24
AMR^a^	41	49
AMR + CPT^a^	9	11
CDDP + VP-16/AMR + CPT^a^	5	6
Cycles		
1/2	11/10	13/12
3/4	12/36	14/43
5/6	2/12	2/14
Response		
Complete response	1	1
Partial response	45	54
Stable disease	10	12
Progression disease	10	12
Not evaluable	17	20
Progression^b^		
Sensitive relapse	23	28
Refractory relapse	60	72
Survival, median, months^c^		
PFS	5.0 (4.5–5.5)	-
OS	9.2 (7.6–10.8)	-
[OS for sensitive relapse]	[19.4 (12.9–25.9)]	-
[OS for refractory relapse]	[6.9 (6.3–7.6)]	-

Note: *ES-SCLC* extensive-stage small cell lung cancer, *CDDP* cisplatin, *CBDCA* carboplatin, *CPT* irinotecan, *VP-16* etoposide, *AMR* amrubicin, *PFS* progression free survival, *OS* overall survival

^a^Including patients in clinical trials

^b^Sensitive relapse: Relapsed three months after completion of the initial therapy, Refractory relapse: Experienced disease progression within three months

^c^(): 95 % confidence interval

### Evaluation of candidate prognostic factors

**Fig. 1:**
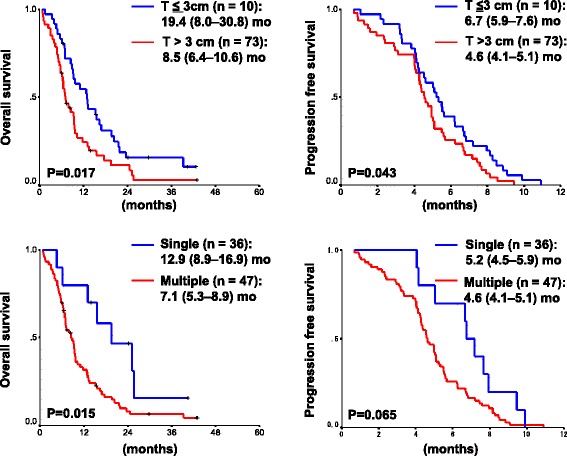
Kaplan–Meier Analysis-based Estimates of Survival Based on Prognostic Factors. 1-1. Comparison of survival between patients having small (≤3 cm) primary tumors (blue) and those having large (>3 cm) primary tumors (red). 1-2. Comparison of survival between patients having single sites of a distant metastasis (blue) and those having multiple (two or more) sites of distant metastases (red). *P*-values were determined by the log-rank test; the number of individuals and the survival times (median (95 % confidence interval), months (mo)) in each group are indicated

Each prognostic factor was assessed using the Kaplan–Meier method (Additional file 1: Table S1). Performance status (PS), primary tumor size based on the TNM classification for lung cancer [16], the number of metastatic sites, and values such as Alb, LDH, sodium and the levels of NSE were significantly associated with the survival in this study. OS was significantly shorter in patients with large tumors (MST: 8.5 mo for >3 cm vs. 19.4 mo for ≤3 cm, *p* < 0.02; Fig. 1(1-1)) and those with multiple metastatic sites (MST: 7.1 mo for multiple sites vs. 12.9 mo for single sites, *p* = 0.015; Fig. 1(1-2)). PFS was also significantly shorter in patients with large tumors (4.6 mo for >3 cm vs. 6.7 mo for ≤3 cm, *p* = 0.043; Fig. 1(1-1)), whereas the patients with multiple metastatic sites had a shorter PFS than those with single metastatic sites (4.6 mo vs. 5.2 mo, *p* = 0.065; Fig. 1(1-2)). The interaction of tumor size and number of metastatic sites was also significant after adjustments for age, gender, PS, LDH levels, and sodium levels using the multiple Cox proportional hazard model analysis (large tumor size: HR 2.44, 95 % CI: 1.05–5.68, *p* = 0.038; multiple metastatic sites: HR 1.81, 95 % CI: 1.08–3.04, *p* = 0.026) (Table 3).

**Table 3:** Multivariate cox proportional hazard model analyses of prognostic factors for ES-SCLC patients treated with chemotherapy

Factors	HR	95 % CI	p
Age ≥75	1.26	0.74–2.14	0.40
Female	2.38	1.11–5.12	0.027
PS 2–4	1.53	0.83–2.81	0.17
Tumor size >3 cm	2.44	1.05–5.68	0.038
Number of metastases ≥2	1.81	1.08–3.04	0.026
LDH high	1.88	0.96–3.67	0.064
Na low	2.50	1.31–4.78	0.006

Note: *ES-SCLC* extensive-stage small cell lung cancer, *HR* hazard ratio, *CI* confidential interval,*P S* performance status

### Intra-thoracic progression after initial chemotherapy

**Fig. 2:**
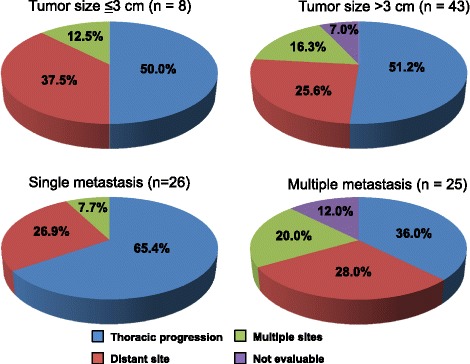
Disease Progression after First-line Chemotherapy in ES-SCLC Patients without Pleural Effusion (*n* = 51). Rates (%) of recurrence patterns based on primary tumor size (≤3 cm vs. >3 cm; 2-1) and the number of metastatic sites (single metastasis vs. multiple metastases; 2-2) at diagnosis are shown. Blue: thoracic progression, Red: metastases to a distant site, Green: metastases to multiple sites including pleural dissemination and carcinomatous lymphangiosis, Purple: not evaluable

The 51 ES-SCLC patients without pleural dissemination were assessed as a targeted subgroup of ES-SCLC patients for TRT combined initial chemotherapy. In these patients, a high rate of thoracic progression after initial chemotherapy was observed in patients with single distant metastasis at diagnosis compared with those with multiple distant metastases (65 % for single sites vs. 36 % for multiple sites, *p* = 0.036), in contrast to primary tumor size (50 % for ≤3 cm vs. 51 % for >3 cm, *p* = 0.95) (Fig. 2, Additional file 2: Table S2).

## Discussion

The prognosis in ES-SCLC patients is poor; the MST without treatment has been reported to range from 2 to 4 mo. Chemotherapy remains the cornerstone of ES-SCLC therapy, although no cure has emerged. Most approaches, including new chemotherapeutic drugs, molecular targeted drugs, or maintenance chemotherapy have not shown improvements successfully [1], and the MST has improved little in recent decades. Local radiotherapy, such as PCI and TRT, indicated that the strategy of combined modality therapy was one of the options for disease control of all stage SCLC.

In European studies, treatment using local radiotherapy, PCI [13] and TRT in addition to PCI [15], suggested an improvement in the survival of ES-SCLC patients. In the first PCI trial, the incidence of symptomatic brain metastases decreased significantly in the PCI group compared with the control group (15 % vs. 40 %) and OS at 1 year improved (27 % vs. 13 %) [13]. However, 75 % of patients had persisting intra-thoracic disease after chemotherapy, and roughly 90 % had intra-thoracic disease progression within the first year, so the randomized trial of TRT in addition to PCI for ES-SCLC patients was assessed [15]. All patients who responded to chemotherapy were targeted for TRT based on the results of 1-year OS (33 % vs. 28 %, *p* = 0.066), 2-year OS (13 % vs. 3 %, *p* = 0.004), PFS (4 vs. 3 mo, *p* = 0.001), the rates of intra-thoracic progression (19.8 % vs. 46.0 %, *p* < 0.0001), and no severe toxic effects between the TRT group and the control group. Although a small number of the ES-SCLC patients (*n* = 5) received TRT during the course of their treatment and the data included limitations such as selection bias in this study, the survival of the patients that received local TRT was longer compared with the other patients (MST: 21.5 mo vs. 9.1 mo, Additional file 3: Figure S1). Local radiotherapy, including TRT, was a standard treatment for patients with LS-SCLC [3], and was an option for those with ES-SCLC selected.

Based on previous evidence, adding TRT to chemotherapy may be a beneficial treatment option for ES-SCLC patients. To use TRT for ES-SCLC patients, the disease should be controlled using systemic chemotherapy and then thoracic lesions to be exposed to radiation should be identified. B. Jeremic et al. performed a randomized study for ES-SCLC patients who had a complete response outside the thorax and at least a partial response within the thorax after three cycles of etoposide and cisplatin [17]. D. Yee et al. conducted a phase II trial of consolidation low dose TRT for ES-SCLC patients who documented objective response to at least one cycle of chemotherapy [18]. B. J Slotman et al. did the phase III randomized trial for ES-SCLC patients who responded to four to six cycles of etoposide and platinum chemotherapy followed by PCI and no clinical evidence of brain, leptomeningeal, or pleural metastases [15]. We considered that a targeted population of ES-SCLC should be selected for the addition of TRT. Our study suggests that the number of metastatic sites (organs) at diagnosis is a prognostic factor predicting intra-thoracic progression.

The concept of “oligometastases” defining a small number of metastases limited to an organ was introduced [19] and revisited [20] for cancer treatments. The term “oligometastases” describes an intermediate state of cancer spreading between localized disease and widespread metastases. The patients with limited metastatic disease, such as liver metastasis from colon cancer, can be cured by metastasis resection [21]. The concept of oligo-recurrence is related to the notion that cancer patients with 1–5 metastatic or recurrent lesions that could be treated by local therapy have controlled primary lesions, and oligometastases and oligo-recurrence may be treated by local therapy such as surgery, radiotherapy, or radiofrequency ablation [22]. Our study shows that ES-SCLC patients with oligometastases should be considered as a population for indication of TRT to improve their survival.

There is a modest association between the overall response rate and MST differences in ES-SCLC trials (*R*^2^ = 0.3314), and stronger association in trials wherein patients with an objective response to the first-line chemotherapy had undergone subsequent PCI (*R*^2^ = 0.6279) [23]. For ES-SCLC, the regimens associated with a lower response rate have a worse outcome [24]. Alternatively, the increased effects of initial treatment may be a predictive factor of survival for patients with ES-SCLC, because the majority of SCLC patients relapse quickly and die of the disease. When we plotted the PFS and OS differences (Additional file 4: Figure S2), a modest relationship was detected between them in the ES-SCLC patients (*R*^2^ = 0.49). The increased effects of initial treatments using combined modalities might lead to survival benefit in the case of aggressive disease.

Our study had several limitations. First, this was a retrospective study from a single institution; the results cannot be completely regarded as definitive. Second, the sample size may not have been sufficient. The patients had received various types of chemotherapy, and most patients with ES-SCLC in this study did not receive clinical TRT. We propose that TRT should be considered for oligometastatic ES-SCLC. However, the results of our study should not cause a treatment change in clinical practice before a more complete evaluation during clinical trials is done.

A therapeutic plateau has been reached with conventional cytotoxic chemotherapy in ES-SCLC. New therapeutic approaches for SCLC need to be developed and predictive biomarkers validated to help individualize therapy. The favorable prognostic factors were limited disease spread, good PS, female gender, young age, the normal level of LDH, response to therapy and PCI [12, 25]. Around 70 % SCLCs are metastatic at diagnosis. Tumor volume should be assessed for combining TRT with systemic chemotherapy to evaluate the radiation field. The presence of circulating tumor cells found in patients with both LS- and ES-SCLC of peripheral venous blood were also predictive and prognostic factors [26]. Biomarker candidates such as tumor marker, circulating tumor cells, and tumor volumes should be evaluated in the clinical trials of TRT combined with chemotherapy for patients with ES-SCLC.

## Conclusion

The present study suggested that large tumor size and multiple distant metastases were poor prognostic factors in ES-SCLC patients treated with chemotherapy. Sixty-five percent of ES-SCLC patients with a single site of distant metastasis had intra-thoracic disease progression after initial chemotherapy. The use of TRT may improve the survival in ES-SCLC patients with limited metastatic disease. TRT should be considered for ES-SCLC patients with single site of distant metastasis. The use of TRT in patients with oligometastatic ES-SCLC should be evaluated in a clinical trial.

## Additional files

Additional file 1: Table S1. Assessment of the Well-Known and Potential Prognostic Factors for Overall Survival in ES-SCLC Patients. (DOC 61 kb) Additional file 2: Table S2. Number of ES-SCLC Patients with Thoracic Progression after Initial Chemotherapy Based on the Primary Tumor Size or the Number of Metastatic Sites. (DOC 92 kb) Additional file 3: Figure S1. Comparison of overall survival between the patients who received thoracic radiotherapy (TRT: blue) and those who did not receive TRT (no TRT: red) in the course of treatment for ES-SCLC (*n* = 83). P-values were determined by the log-rank test; the number of individuals and overall survival times (median (95% confidence interval), months (mo)) in each group are indicated. (DOC 61 kb) Additional file 4: Figure S2. Association between Progression Free Survival and Overall Survival for ES-SCLC Patients in This Study. Blue circles represent individual patients with ES-SCLC. (DOC 92 kb)

## Abbreviations

CI: confidential interval
ES: extensive-stage
LS: limited-stage
mo: months
MST: median survival time
OS: overall survival
PCI: prophylactic cranial irradiation
PFS: progression free survival
SCLC: small cell lung cancer
TRT: thoracic radiotherapy
