# Supplementary figures and images for: Prognostic factors affecting the risk of thoracic progression in extensive-stage small cell lung cancer
Source: BMC Cancer. 2016 Mar 8;16:197. doi: 10.1186/s12885-016-2222-4 (PMC4782389; doi:10.1186/s12885-016-2222-4)

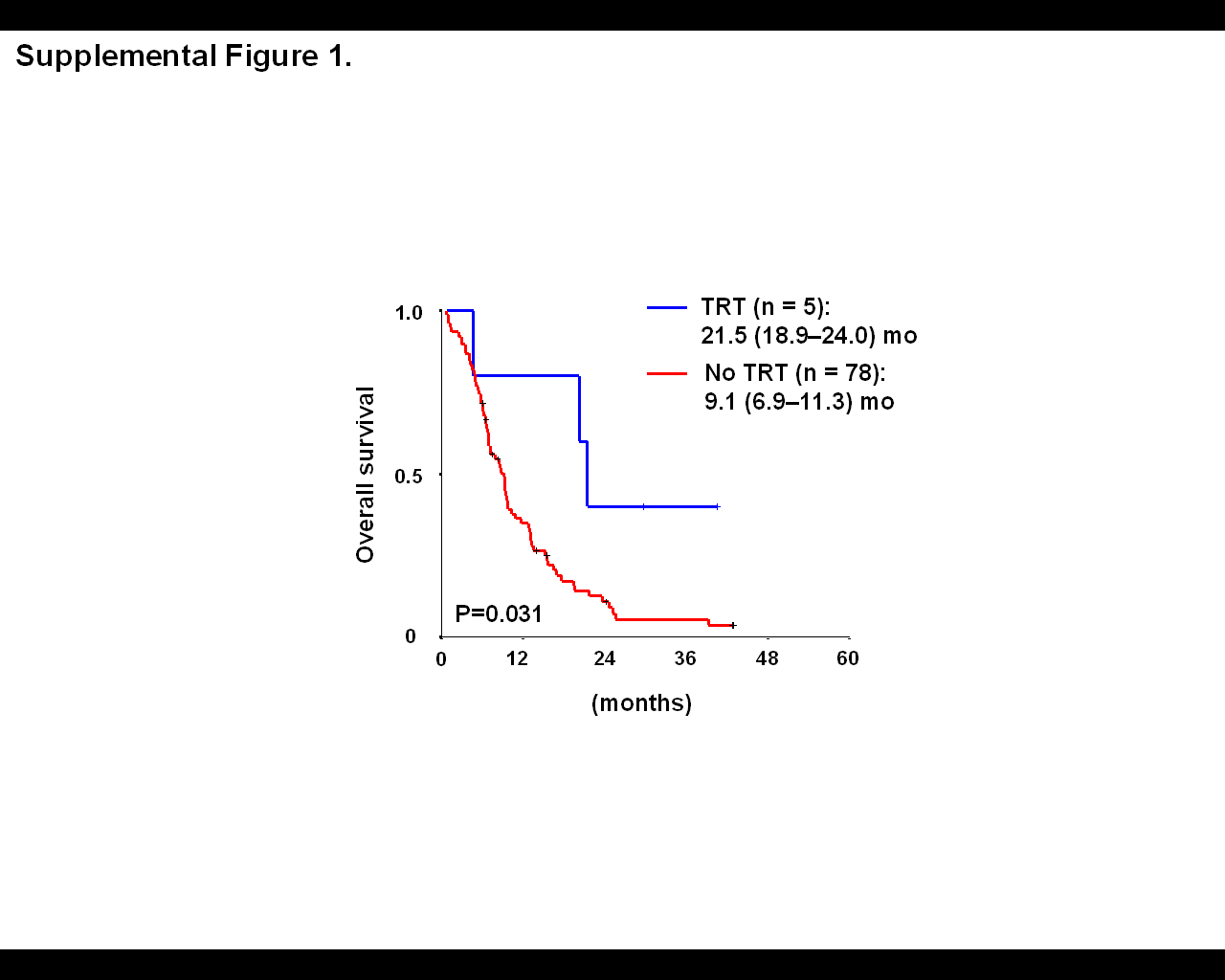

Supplement: Additional file 1: Table S1. — Assessment of the Well-Known and Potential Prognostic Factors for Overall Survival in ES-SCLC Patients. (DOC 61 kb) [file 12885_2016_2222_MOESM1_ESM.doc]

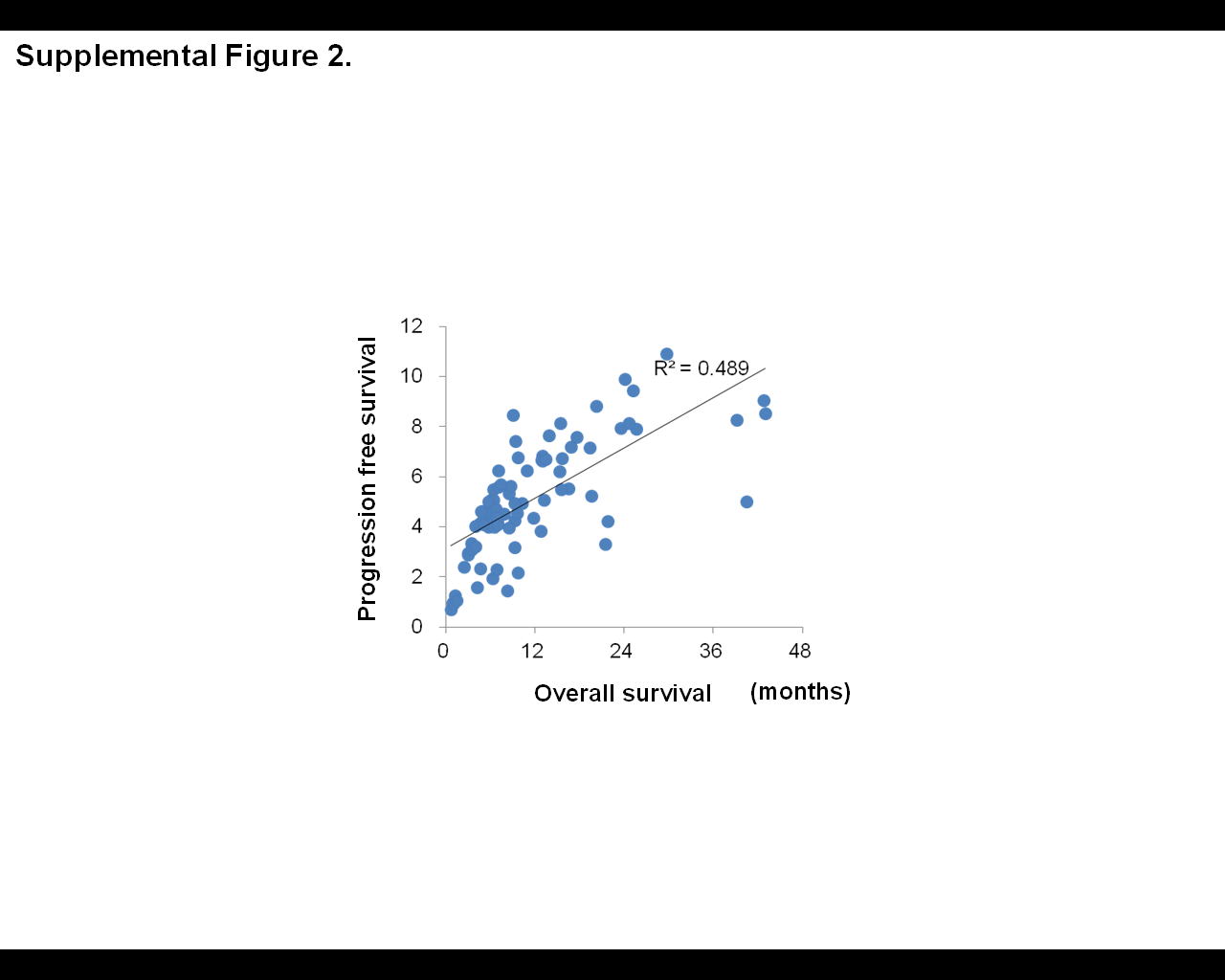

Supplement: Additional file 2: Table S2. — Number of ES-SCLC Patients with Thoracic Progression after Initial Chemotherapy Based on the Primary Tumor Size or the Number of Metastatic Sites. (DOC 92 kb) [file 12885_2016_2222_MOESM2_ESM.doc]

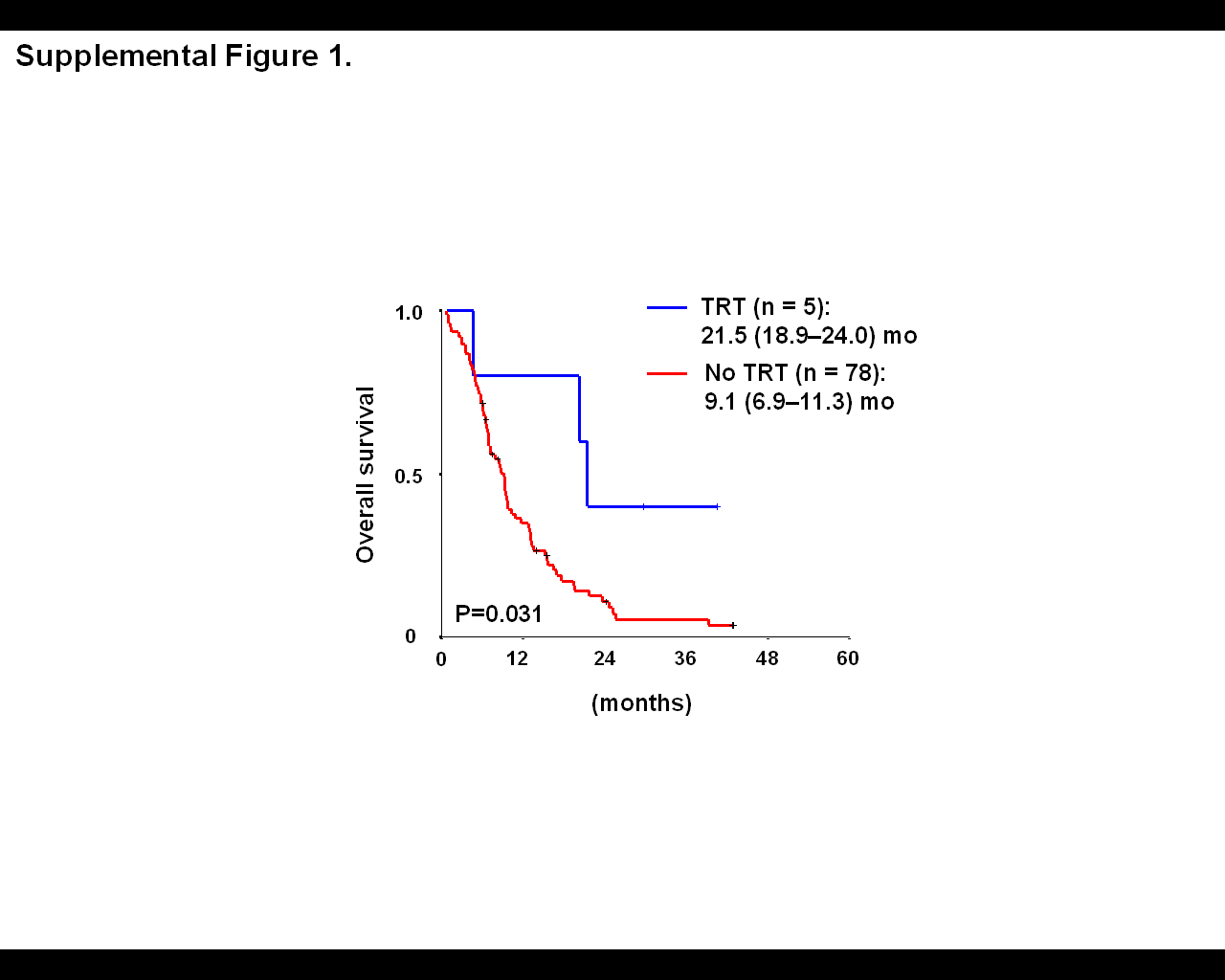

Supplement: Additional file 3: Figure S1. — Comparison of overall survival between the patients who received thoracic radiotherapy (TRT: blue) and those who did not receive TRT (no TRT: red) in the course of treatment for ES-SCLC (n = 83). P-values were determined by the log-rank test; the number of individuals and overall survival times (median (95% confidence interval), months (mo)) in each group are indicated. (DOC 61 kb) [file 12885_2016_2222_MOESM3_ESM.doc]

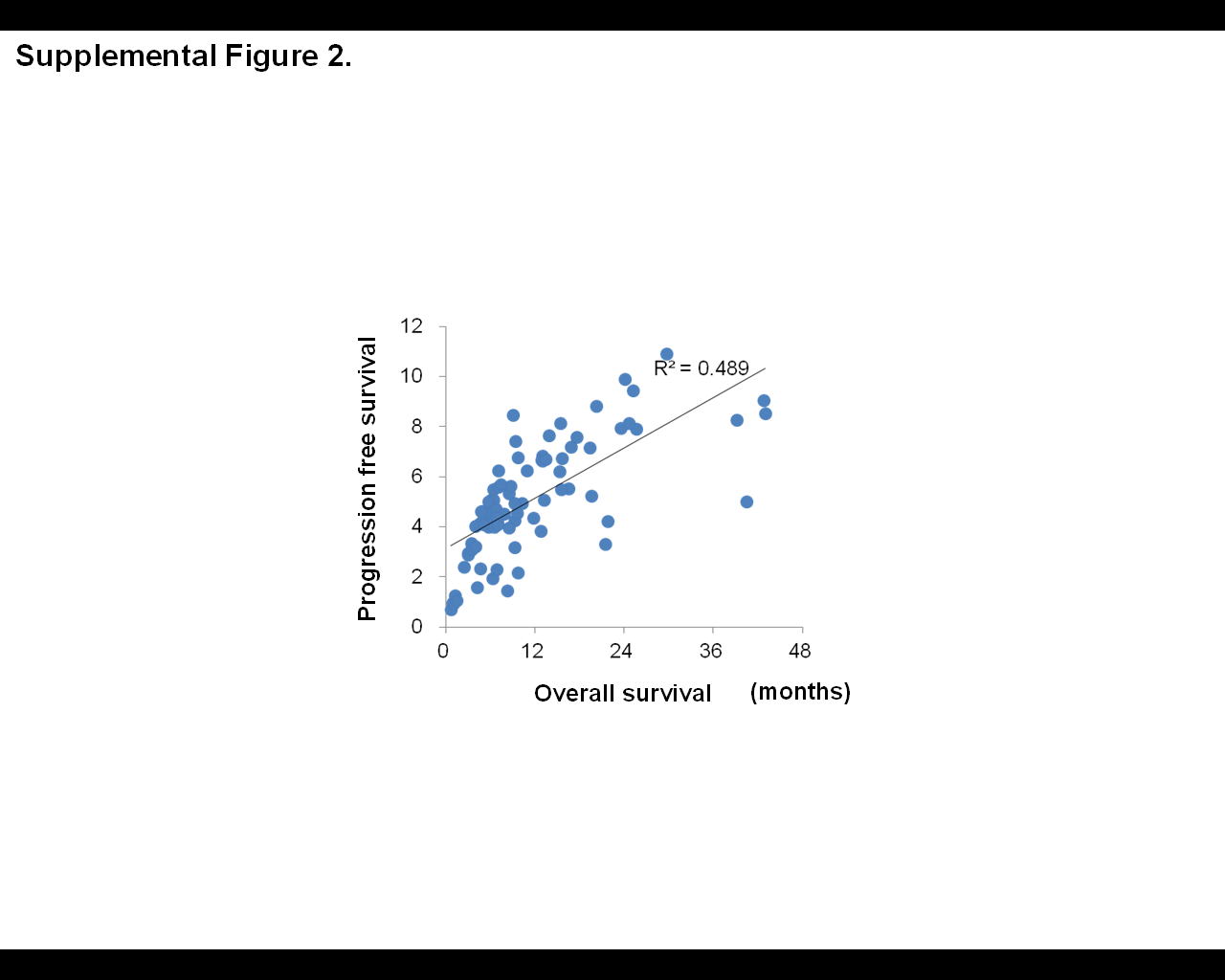

Supplement: Additional file 4: Figure S2. — Association between Progression Free Survival and Overall Survival for ES-SCLC Patients in This Study. Blue circles represent individual patients with ES-SCLC. (DOC 92 kb) [file 12885_2016_2222_MOESM4_ESM.doc]
